# Manualising the induction of higher trainees in psychiatry for North Wales: The CiSGC Guide (“Croeso i Seiciatreg Gogledd Cymru”)

**DOI:** 10.1192/bjo.2021.572

**Published:** 2021-06-18

**Authors:** Jawad Raja, Jiann Lin Loo, Rajvinder Sambhi, Somashekara Shivashankar

**Affiliations:** 1 Ysbyty Maelor Wrecsam, Betsi Cadwaladr University Health Board; 2 Cardiff and Vale University Health Board

## Abstract

**Aims:**

There is a significant period of adjustment for new higher trainees in psychiatry given the presence of inter-trust differences in the National Health Services (NHS). It may take some time for a trainee to become familiar with the new administrative system and workflow of the new environment, which may be even longer for an international medical graduate (IMG). Although there is an existing induction system, having a written structured manual will assist the trainees to get through this process more easily. Hence, this Quality Improvement Project (QIP) outlined the creation of an induction manual that serves as a starter pack to facilitate the settling-in process of new North Wales higher trainees in psychiatry, i.e. the “Croeso i Seiciatreg Gogledd Cymru” (CiSGC) guide (means Welcome to North Wales Psychiatry in Welsh).

**Method:**

The induction manual was initially drafted by the authors based on the available printed policies and information online. Further input and from different stakeholders were obtained to triangulate and enrich the manual. Specific links and further references were included in the manual for the reference of prospective manual users. Authors’ contact details were included for any further clarification, suggestions or input.

**Result:**

The manual consisted of four sections: A) General Process before, during and after Reporting Duty, B) Trainees’ Duty, 3) Speciality-specific Guidance, and 4) Health Board-related Information. The General Process section covered the visa-related information, post-acceptance paperwork process, access to email and hospital informative system, medical practice-related issues (including section 12(2) approval and medical indemnity). The Trainees’ Duty section briefed on time-tabling and clinical duty. The Specialty-specific Guide provided important information related to training. Lastly, the section of Health Board-related Information highlighted the administrative structure of the NHS Health Board, important contact numbers, link to information. Specialty specific sections were created for general adult psychiatry and old age psychiatry as there is no other higher training of psychiatry in North Wales at the moment. Further sections in the pipeline include substance misuse and liaison psychiatry.

**Conclusion:**

This induction manual is neither prescriptive nor exhaustive. It serves as a generic reference to facilitate new trainees in their adjustment process. Further review and revision will be conducted before every induction process to ensure the information is up-to-date and incorporating new input from the trainees.

